# Nickel(II) Complex of Polyhydroxybenzaldehyde N4-Thiosemicarbazone Exhibits Anti-Inflammatory Activity by Inhibiting NF-κB Transactivation

**DOI:** 10.1371/journal.pone.0100933

**Published:** 2014-06-30

**Authors:** Hana Bashir Shawish, Wan Ying Wong, Yi Li Wong, Sheng Wei Loh, Chung Yeng Looi, Pouya Hassandarvish, Alicia Yi Ling Phan, Won Fen Wong, Hao Wang, Ian C. Paterson, Chee Kwee Ea, Mohd Rais Mustafa, Mohd Jamil Maah

**Affiliations:** 1 Department of Chemistry, Faculty of Science, University of Malaya, Kuala Lumpur, Malaysia; 2 Institute of Biological Sciences at the Faculty of Science, University of Malaya, Kuala Lumpur, Malaysia; 3 Department of Pharmacology, Faculty of Medicine, University of Malaya, Kuala Lumpur, Malaysia; 4 Department of Medical Microbiology at the Faculty of Medicine, University of Malaya, Kuala Lumpur, Malaysia; 5 School of Pharmacy, Ningxia Medical University, Yinchuan, Ningxia, P. R. China; 6 Department of Oral Biology and Biomedical Sciences, and Oral Cancer Research and Coordinating Centre, Faculty of Dentistry, University of Malaya, Kuala Lumpur, Malaysia; University of Kansas School of Medicine, United States of America

## Abstract

**Background:**

The biological properties of thiosemicarbazone have been widely reported. The incorporation of some transition metals such as Fe, Ni and Cu to thiosemicarbazone complexes is known to enhance its biological effects. In this study, we incorporated nickel(II) ions into thiosemicarbazone with N4-substitution groups H_3_L (H; H_3_L^1^, CH_3_; H_3_L^2^, C_6_H_5_; H_3_L^3^ and C_2_H_5_; H_3_L^4^) and examined its potential anti-inflammatory activity.

**Methodology/Principal Findings:**

Four ligands (1–4) and their respective nickel-containing complexes (5–8) were synthesized and characterized. The compounds synthesized were tested for their effects on NF-κB nuclear translocation, pro-inflammatory cytokines secretion and NF-κB transactivation activity. The active compound was further evaluated on its ability to suppress carrageenan-induced acute inflammation *in vivo*. A potential binding target of the active compound was also predicted by molecular docking analysis.

**Conclusions/Significance:**

Among all synthesized compounds tested, we found that complex [Ni(H_2_L^1^)(PPh_3_)]Cl (**5**) (complex **5**), potently inhibited IκBα degradation and NF-κB p65 nuclear translocation in LPS-stimulated RAW264.7 cells as well as TNFα-stimulated HeLa S3 cells. In addition, complex **5** significantly down-regulated LPS- or TNFα-induced transcription of NF-κB target genes, including genes that encode the pro-inflammatory cytokines TNFα, IFNβ and IL6. Luciferase reporter assays confirmed that complex **5** inhibited the transactivation activity of NF-κB. Furthermore, the anti-inflammatory effect of complex **5** was also supported by its suppressive effect on carrageenan-induced paw edema formation in wild type C57BL/6 mice. Interestingly, molecular docking study showed that complex **5** potentially interact with the active site of IKKβ. Taken together, we suggest complex **5** as a novel NF-κB inhibitor with potent anti-inflammatory effects.

## Introduction

Nickel (Ni) is an essential trace element for bacteria, plants, animals and humans. The concentration of nickel in human tissue is relatively low (1 µg/L) compared to zinc, iron and copper (100 µg/L) [1]. Studies to investigate the biological functions of nickel have expanded dramatically following the discovery of nickel as an active site metal in the jack bean urease by Zerner and his co-workers in 1975. We now know that a number of enzymes depend on nickel, including urease, NiFe hydrogenase, CO dehydrogenase, acetyl-CoA synthase, methyl-Coenzyme M reductase, glyoxalase I, acireductone dioxygenase, and nickel containing superoxide dismutase [2]. These nickel-containing enzymes are utilized in a diverse range of biological reactions attributed to nickel's flexibility including varied coordination numbers, geometries and oxidation states.

Thiosemicarbazone complexes were discovered a few decades ago and they are now attracting growing interest because of their pharmacological properties including antiviral, antibacterial, antimalarial [3], [4] and anticancer effects [5]. Thiosemicarbazone compounds are the products of condensation reactions of a suitable aldehyde or ketone with thiosemicarbazide or substituted thiosemicarbazide at the N4 position. The biological activities of thiosemicarbazone can be modified or enhanced by the linkage to important metal ions such as Fe, Ni, Cu and Zn [6]. For example, nickel complexes of thiosemicarbazone have been shown to possess anti-proliferative activity in a few cancer cell lines [3], [7], [8].

Inflammation is a physiological response to injury and infectious agents, such as viruses and microbes [9]. Studies revealed the involvement of inflammation in the progression of various diseases, especially cancers, which supports the hypothesis by Rudolf Virchow that malignant cancerous cells can originate from sites of chronic inflammation [9]–[11]. Transcription factor NF-κB (nuclear factor-kappa-light-chain-enhancer of activated B cells) plays an indispensable role in the pathogenesis of acute or chronic inflammation [12]. The NF-κB family consists of five members, p65 (RelA), RelB, c-Rel, p50 and p52 that associate with each other to form homo- or heterodimeric complexes. The NF-κB molecules are retained in cytoplasm by interaction with inhibitor of kappa-B (IκB). Upon stimulation, IκB will be degraded and dissociated from NF-κB, which enables NF-κB migration from the cytoplasm into the nucleus. Once activated by ligands, such as LPS or TNFα, NF-κB will trigger the transcription of numerous genes involved in pro-inflammatory responses [13]–[17]. Thus, targeting or inhibiting NF-κB transactivation has potential therapeutic value, as NF-κB is the key molecule involved in inflammatory pathogenesis.

Though the versatility binding modes of thiosemicarbazone to nickel ion have been reported, the biological effect of various N4-substituted groups is unknown. In the present study, four thiosemicarbazone derivatives of 2,3-dihydroxybenzaldehyde, differing in the substituent on N4 of the thiosemicarbazone (H; H_3_L^1^, CH_3_; H_3_L^2^, C_6_H_5_; H_3_L^3^ and C_2_H_5_; H_3_L^4^), were synthesized and their anti-inflammatory effects were examined. We presented evidence to show that one of the synthesized compounds potently inhibited inflammation *in vitro* and *in vivo*.

## Materials and Methods

### Synthesis of thiosemicarbazone ligands and complexes

Triphenylphosphine was purchased from Merck, nickel(II) [Ni(II)] chloride hexahydrate, potassium thiocyanate was from Sigma-Aldrich and reagents were of analytical grade and used without further purification. [NiCl_2_(PPh_3_)_2_] was prepared according to the published procedure [18]. The thiosemicarbazone ligands H_3_L(**1–4**) were prepared following the published procedure [19] by reaction of 2,3-dihydroxybenzaldehyde with thiosemicarbazide (H_3_L^1^; ligand **1**), 4-methyl-3-thiosemicarbazide (H_3_L^2^; ligand **2**), 4-phenyl-3-thiosemicarbazide (H_3_L^3^; ligand **3**) and 4-ethyl-3-thiosemicarbazide (H_3_L^4^; ligand **4**) in a 1: 1 molar ratio, under standard reflux conditions. Complexes (**5–8**) were synthesized according to the published works [20], [21]. Complexes [Ni(H_2_L^1^)(PPh_3_)]Cl (**5**), [Ni(H_2_L^2^)(PPh_3_)] (**6**), [Ni(HL^3^)(PPh_3_)] (**7**) and [Ni(HL^4^)(PPh_3_)] (**8**) were also named as complex **5**, **6**, **7** and **8** below. For biological testing, stock solutions (10 mg/ml) of these compounds were prepared in 10% v/v aqueous dimethylsulfoxide (DMSO) (Fisher chemicals).

### IR and NMR spectra measurements

Infrared (IR) spectra were recorded as KBr pellets in the frequency range of 400–4000 cm^−1^ by using a Perkin-Elmer Spectrum RX-1 spectrophotometer. Nuclear magnetic resonance (NMR) spectra were recorded in deuterated DMSO-d_6_ on ECA 400 MHz instrument. Elemental analyses were performed on a Pelkin-Elmer Analyst 400.

### X-ray crystallography

Recrystallization of complex [Ni(HL^3^)(PPh_3_)] (**7**) from a mixture of dimethylformamide/ethanol afforded plate purple crystals whereas block red crystals of the complex [Ni(HL^4^)(PPh_3_)] (**8**) were obtained from its methanol mother liquor. Data were collected on a Bruker SMART APEX CCD area detector diffractometer, equipped with a highly-oriented pyrolytic graphite crystal incident beam monochromator and a molybdenum Kα (λ = 0.71073 Å). The APEX2 software was used for data acquisition and the SAINT software for cell refinement and data reduction [22]. SADABS software was used for Absorption data corrections [23]. The structures were solved and refined by SHELXL97 package [24]. Molecular graphics were drawn by using ORTEP [25].

### Cell culture

RAW264.7, HeLa S3 and K562 cells were purchased from ATCC. KCL22 cells was as previously described [26]. RAW264.7, K562 and KCL22 cells were cultured in RPMI, while HeLa S3 and 293T-luc cells were cultured in DMEM. Both media were supplemented with 10% fetal bovine serum, penicillin G (100 µg/ml), and streptomycin (100 µg/ml). Cells were maintained at 37°C with 5% CO_2_ in a humidified incubator.

### Cell proliferation assay

A total number of 1.0×10^4^ cells per well were seeded into a 96-well plate and incubated overnight at 37°C in 5% CO_2_. The next day, the cells were treated with a two-fold serial dilution of compounds. MTT assays were performed as described [27]. After 48 h, (4,5-dimethylthiazol-2-yl-2,5-diphenyltetrazoliumbromide) was added at 2 mg/ml. After 3 h of incubation at 37°C in 5% CO_2_, DMSO was added to dissolve the formazan crystals. The plates were then read in Chameleon multitechnology microplate reader (Hidex, Turku, Finland) at 570 nm absorbance. The ratio of the absorbance of treated cells to the absorbance of DMSO-treated control cells was determined as percentage cell viability. The concentration of compounds which resulted in a 50% reduction in viability was defined as the IC_50_.

### ORAC antioxidant assay

ORAC assay was performed based on reported procedures [28] with slight modifications. Chemicals used such as fluorescein sodium salt, AAPH (2,2′-Azobis(2-methylpropionamidine) dihydrochloride), quercetin dehydrate and trolox ((±)-6-Hydroxy-2,5,7,8-tetramethylchromane-2-carboxylic acid) were purchased from Sigma-Aldrich. Compounds were diluted to a final concentration of 100 µg/ml, with total reaction volume of 200 µl. The assay was performed in a 96-well black microplate, with 25 µl of samples, standard (trolox), blank (solvent/PBS) or positive control (quercetin). Subsequently, 150 µl of working fluorescein solution was added to each well. The plate was incubated at 37°C for at least 5 min. Then, 25 µl of AAPH working solution was then added to the wells to make a total volume of 200 µl. Fluorescence was read in Chameleon multitechnology microplate reader (Hidex, Turku, Finland) with excitation wavelength of 485 nm and emission wavelength of 538 nm. Data were collected every 2 min for a duration of 2 h, and were analyzed by calculating the differences of area under fluorescence decay curve (AUC) of samples and blank. The values were expressed as Trolox equivalent (TE).

### Immunofluorescence staining

NF-κB Activation Kits (Thermo Scientific) were used as previously described [29]. Cells were pretreated with compounds for 4 h before being stimulated with lipopolysaccharide (LPS) ortumour necrosis factor α (TNFα) for 1 h. Cells were then fixed and permeabilized before probing with NF-κB p65 antibody for 1 h. Staining solution (containing DyLight 488 Goat Anti-Rabbit and Hoechst dye) was then added and incubated for 1 h. The plate with stained cells was evaluated using a Cellomics ArrayScan HCS Reader (Cellomics, PA, USA). Data were captured, extracted and analyzed with ArrayScan II Data Acquisition and Data Viewer version 3.0 (Cellomics).

### Immunoblot analysis

Cells were pretreated with or without of complex **5** (25 µg/ml or 50 µg/ml) for 4 h before treatment with LPS (10 ng/ml) or TNFα (10 ng/ml), for the indicated times. The cytoplasmic extracts were prepared by using the hypotonic lysis buffer (10 mM Tris, pH 7.5; 1.5 mM MgCl_2_; 10 mM KCl; 0.5 mM DTT; 0.5 mM PMSF; 1× Protease Inhibitor, 0.1% NP40) followed by incubation on ice for 20 min and centrifugation at 3k rpm at 4°C for 10 min. The supernatants were collected as cytoplasmic extracts. The nuclear pellets were resuspended in the whole cell lysis buffer (25 mM Tris, pH7.5; 420 mM NaCl; 1.5 mM MgCl_2_; 0.2 mM EDTA; 25% Glycerol; 0.5 mM DTT; 0.5 mM PMSF; 1× Protease Inhibitor). The nuclear extracts were then collected by centrifugation at 15 krpm at 4°C for 10 min. Immunoblotting was performed using antibodies against PARP (F-2, N-20), HSP90a (C-20), p65 (C-20), IκBα (C-21) and p-IκBα from Santa Cruz Biotech.

### Quantitative PCR analysis

Cells pretreated with or without of complex **5** for 4 h were incubated with LPS or TNFα for 4 h. Total RNAs were isolated with Thermo Scientific GeneJET RNA Purification Kit. Complimentary DNAs were synthesized and Quantitative PCR was performed with 2× SYBR Green PCR Master mix (Thermo Scientific) and run on the Bio-Rad CFX 96 Real-Time PCR System. All data were then normalized to L32. The sequences of the primers are listed in Supporting Information Table S1.

### Luciferase reporter assay

293T cells were transduced with a lentivirus carrying luciferase reporter driven by NF-κB enhancer found in immunoglobulin kappa light chain gene to prepare 293T-luc cells. The 293T-luc cells were pretreated with or without of complex **5** (12.5 µg/ml, 25 µg/ml, 50 µg/ml) for 4 h. The cells were later stimulated with TNFα (10 ng/ml) for 12 h. Cells were scraped, collected into 1.5 ml tubes and centrifuged at 1.5 k rpm 4°C for 5 min before removing the culture medium. Cell pellets were resuspended in 50 µl luciferase lysis buffer (100 mM Sodium Phosphate buffer, pH7.8; 8 mM MgCl_2_; 1% Triton X-100; 15% glycerol; 1 mM DTT). A total volume of 50 µl of luciferase substrate (containing 1 mM ATP; 0.25 mM luciferin; 1% BSA) was added to each well on a white 96-well plate containing 20 µg lysate. Luciferase activity was measured in 96-well plate with a VarioskanFlash microplate reader (Thermo Scientific).

### In vivo inflammatory assay

Male C57BL/6 mice at age 8–12 weeks old were from Jackson Laboratories, USA. Mice were administrated intraperitoneally (i.p.) with two different doses of complex **5** (2.5 mg/kg and 5 mg/kg). Negative control groups were i.p. injected with 200 µl of vehicle (50% v/v ethanol). A standard drug 2 mg/kg dexamethasone (Sigma) was included in the study. After 1 h, paw edema was induced by injecting 50 µl of 1% carrageenan (Sigma) through the plantar tissue at the right hind paw of each mouse. The thickness of right paw of each mouse was measured at 1, 3 and 5 h after carrageenan was administered. Four animals per group were tested (n = 4). Quantification of right hind paw thickness were analyzed for statistical significance using Student's *t*-test. Animals were housed in individually ventilated cages in specific pathogen free facility, Animal Experimental Unit, University of Malaya. All efforts were made to minimize animal suffering and the number of animals used. At the end of the experiment, mice were euthanized by CO_2_ asphyxiation. Animal work was approved by the Faculty of Medicine Animal Care and Use Committee (FOMIACUC) at University of Malaya and reported according to ARRIVE guidelines.

### Molecular docking

GOLD (v 5.1; Genetic Optimization for Ligand Docking) [30] was used to dock compounds into IκB kinase β (IKKβ). The bound inhibitor (compound 1) was used to indicate the binding site (all protein atoms within 5.0 Å) within the kinase domain of IKKβ (PDB 3RZF) [31]. The crystal structure of IKKβ was treated by Amber 12 with amber **ff12SB** force field and all hydrogen atoms were added. GOLD was used to dock each ligand 10 times, starting each time from a different random population of ligand orientations and using the default automatic genetic algorithm parameter settings. All torsion angles in each compound were allowed to rotate freely. For the bound Ni atom, Zn was substituted as the best surrogate for Ni [32].

## Results

### Spectroscopic characterization

**Figure 1:**
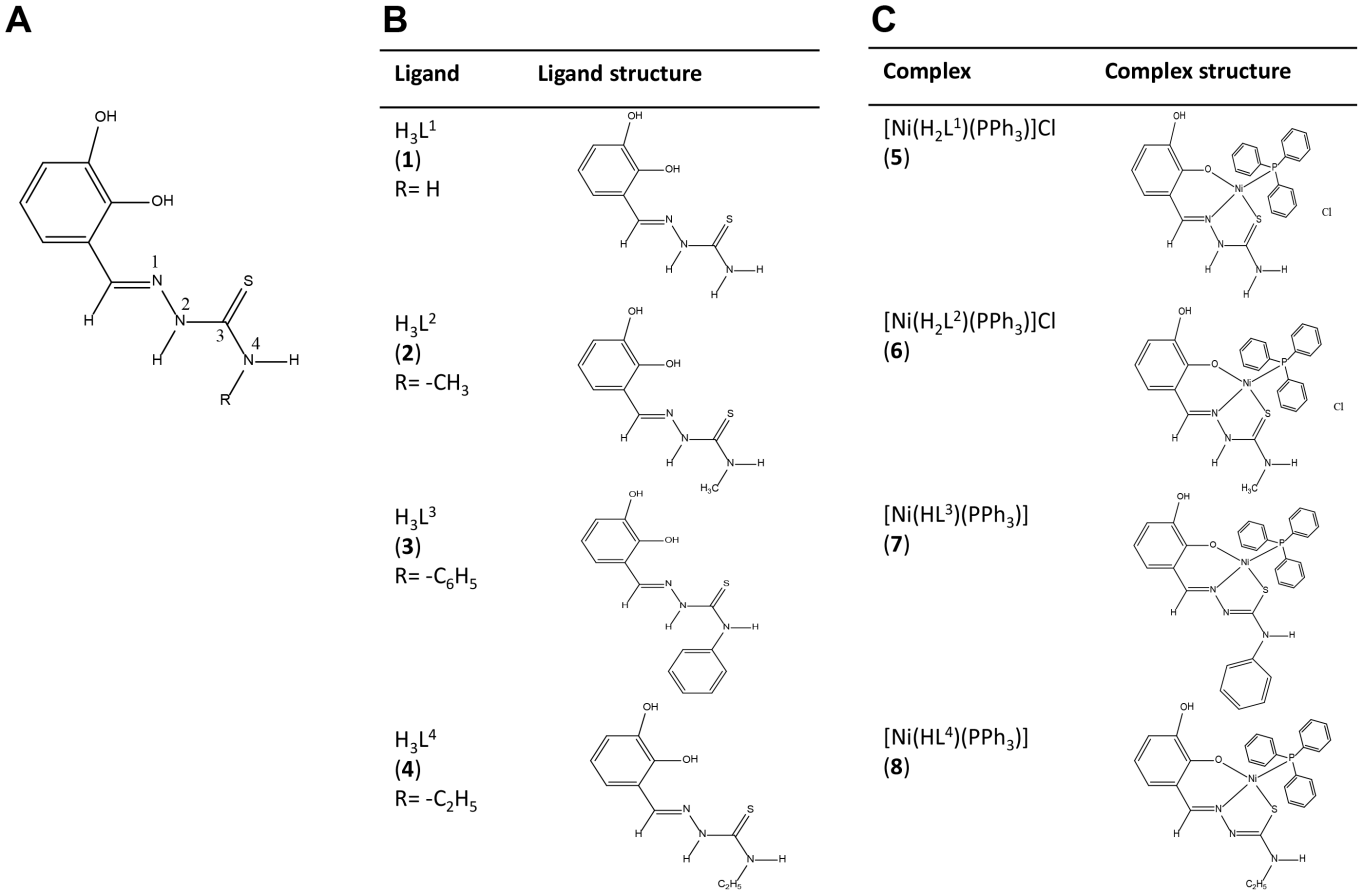
Chemical structures of thiosemicarbazone complexes. (A) 2,3-dihydroxybenzaldehyde –N4-subsituted thiosemicarbazone, the backbone structure of the complexes. (B) Chemical structures of thiosemicarbazone complexes with N4 substituted with –H, -CH_3_, - C_6_H_5_ and -C_2_H_5_ groups. These complexes were regarded as ligands. (C) Chemical structures of ligands with additional triphenyl phosphine group as coligand.

**Figure 2:**
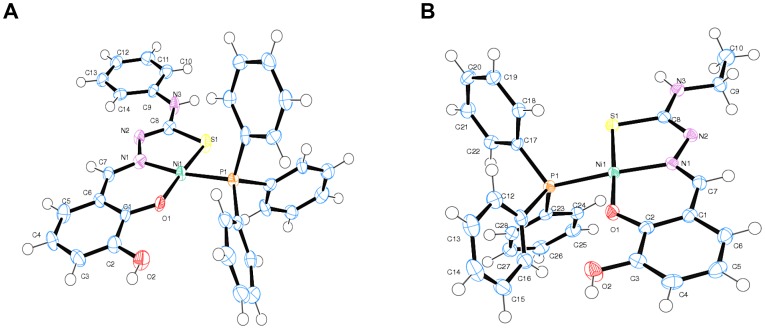
Crystal structures of two of the complexes. (A) ORTEP diagram for complex [Ni(HL^3^)(PPh_3_)] (**7**). (B) ORTEP diagram for compound [Ni(HL^4^)(PPh_3_)] (**8**).

Four complexes and four respective ligands were synthesized and the chemical structures were as depicted (Figure 1). The most significant IR bands for nickel(II) phosphine mixed complexes of 2,3-dihydroxybenzaldehyde-N4-subsituted thiosemicarbazone were collected with their tentative assignments (Table 1). The crystal structures of complexes **5** and **6** have been described previously [20], [21]. The title complexes **7** and **8** crystallized into orthorhombic crystal system. The molecular structures of [Ni(HL^3^)(PPh_3_)] (**7**) and [Ni(HL^4^)(PPh_3_)] (**8**) were as shown (Figure 2A and 2B), while the selected bond lengths and bond angles were as summarized (Table 2).

**Table 1:** IR spectral assignments for the Ni(II) thiosemicarbazone ligands and their Ni(II)-phosphine complexes.

Complex	ν(C = N)	ν (C = S)/ν (C-S)	ν(C-O)	ν(Ni-S)	ν(Ni-O)	ν(Ni-N)	Bands due To PPh_3_
**H_3_L^1^ (1)**	1611	1342 824	1279	-	-	-	-
**[Ni(H_2_L^1^)(PPh_3_)]Cl (5)**	1622	1332 777	1231	483	514	534	1434 1057
							694
**H_3_L^2^ (2)**	1543	1389 853	1290	-	-	-	-
**[Ni(H_2_L^2^)(PPh_3_)]Cl (6)**	1622	1375 781	1279	486	510	532	1437 1048 696
**H_3_L^3^ (3)**	1542	1389 850	1289	-	-	-	-
**[Ni(HL^3^)(PPh_3_)] (7)**	1590	1308 734	1268	474	511	565	1457 1074 693
**H_3_L^4^ (4)**	1542	1384 807	1307	-	-	-	-
**[Ni(HL^4^)(PPh_3_)] (8)**	1592	1308 740	1246	-	510	531	1458 1050 695

**Table 2:** Selected bond lengths (Å) and angles (°) for complex 8.

Bond lengths		Bond angles	
Ni1—O1	1.8510(12)	O1—Ni1—N1	94.93(6)
Ni1—N1	1.8961(14)	O1—Ni1—S1	173.72(4)
Ni1—P1	2.2059(5)	N1—Ni1—S1	87.03(4)
Ni1—S1	2.1488(5)	O1—Ni1—P1	88.61(4)
S1—C8	1.7529(18)	N1—Ni1—P1	171.55(4)
P1—C17	1.8176(17)	S1—Ni1—P1	90.261(18)
P1—C11	1.8264(18)	C8—S1—Ni1	95.92(6)
P1—C23	1.8249(17)	C7—N1—N2	112.62(14)
O1—C2	1.321(2)	C7—N1—Ni1	125.76(12)
O2—C3	1.368(2)	N3—C8—S1	118.33(13)
O2—H1	0.8400	N2—C8—S1	123.33(13)
N1—C7	1.297(2)	N2— C8— N3	118.32(16)
N1—N2	1.400(2)	C17— P1 —Ni1	114.97(6)
N2—C8	1.299(2)	C11 —P1 —Ni1	114.64(6)
N3—C8	1.359(2)	C23 —P1— Ni1	112.34(6)

### Cytotoxic effect of nickel complexes of thiosemicarbazone

To examine whether synthesized Ni(II) thiosemicarbazone ligands or Ni(II)-phosphine complexes were cytotoxic, we treated RAW264.7 (leukemic monocyte macrophage) and HeLa S3 (cervical adenocarcinoma) cells with these compounds for 48 h. The cell viability was determined with MTT assays [33]. These compounds exerted low to moderate cytotoxicity on RAW264.7 cells with an IC_50_ ranging from 19.37 to 56.31 µg/ml (Table 3). The proliferation of HeLa S3 cells was unaffected by most of the compounds, with IC_50_ more than 100 µg/ml, suggesting better tolerability to these compounds in HeLa S3 cells.

**Table 3:** Cell proliferation assay.

RAW264.7		HeLa S3	
Complex	IC_50_ (µg/ml)	Complex	IC_50_ (µg/ml)
H_3_L^1^ (**1**)	22.84±1.67	H_3_L^1^ (**1**)	>100
H_3_L^2^ (**2**)	27.84±0.96	H_3_L^2^ (**2**)	>100
H_3_L^3^ (**3**)	31.48±1.82	H_3_L^3^ (**3**)	>100
H_3_L^4^ (**4**)	19.37±1.07	H_3_L^4^ (**4**)	87.19±1.81
[Ni(H_2_L^1^)(PPh_3_)]Cl (**5**)	28.63±2.24	[Ni(H_2_L^1^)(PPh_3_)]Cl (**5**)	29.31±1.10
[Ni(H_2_L^2^)(PPh_3_)]Cl (**6**)	37.73±3.62	[Ni(H_2_L^2^)(PPh_3_)]Cl (**6**)	77.55±1.56
[Ni(HL^3^)(PPh_3_)] (**7**)	42.44±4.17	[Ni(HL^3^)(PPh_3_)] (**7**)	>100
[Ni(HL^4^)(PPh_3_)] (**8**)	56.31±3.86	[Ni(HL^4^)(PPh_3_)] (**8**)	>100

IC_50_ values of Ni(II) thiosemicarbazone ligands and their Ni(II)-phosphine complexes on RAW 264.7 cells and HeLa S3 cells at 48 h treatment.

### Complex 5 efficiently inhibits LPS-induced NF-κB translocation

**Figure 3:**
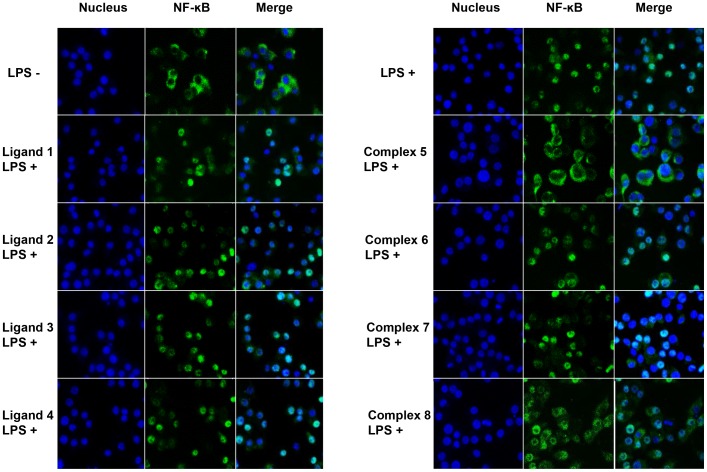
Effect of N4-substituteted thiosemicarbazone complexes on LPS-induced NF-κB nuclear transloction in RAW264.7 macrophage cells. Staining of Hoechst (nucleus) and NF-κB in RAW264.7 cells. Cells were pre-treated with 25 µg/ml of compounds for 4 h, followed by LPS stimulation for 1 h.

To investigate the anti-inflammatory effect, we treated RAW264.7 cells with Ni(II) thiosemicarbazone ligands or Ni(II)-phosphine complexes before stimulating them with LPS (a potent inducer and activator of NF-κB). NF-κB acts as a central mediator of inflammatory responses and compounds that inhibit NF-κB activation are potential anti-inflammatory agents. To evaluate this, we performed immunofluorescence staining of NF-κB p65. In the absence of LPS, we showed that NF-κB p65 remained in the cytoplasm of RAW264.7 cells (Figure 3). In response to LPS stimulation, NF-κB p65 translocated from cytoplasm into the nucleus, implying NF-κB activation (Figure 3). Next, we tested all compounds using this model and found that complex **5** efficiently inhibited LPS-mediated NF-κB p65 nuclear translocation at 25 µg/ml (Figure 3). Of note, the ligand of complex **5** (Ligand **1**, H_3_L^1^) and other compounds did not affect NF-κB translocation at similar concentration. Moreover, we observed that complex **6–8** required higher concentration (>50 µg/ml) to elicit similar inhibitory effect (data not shown).

### Complex 5 inhibits NF-κB translocation by interrupting IκB degradation

**Figure 4:**
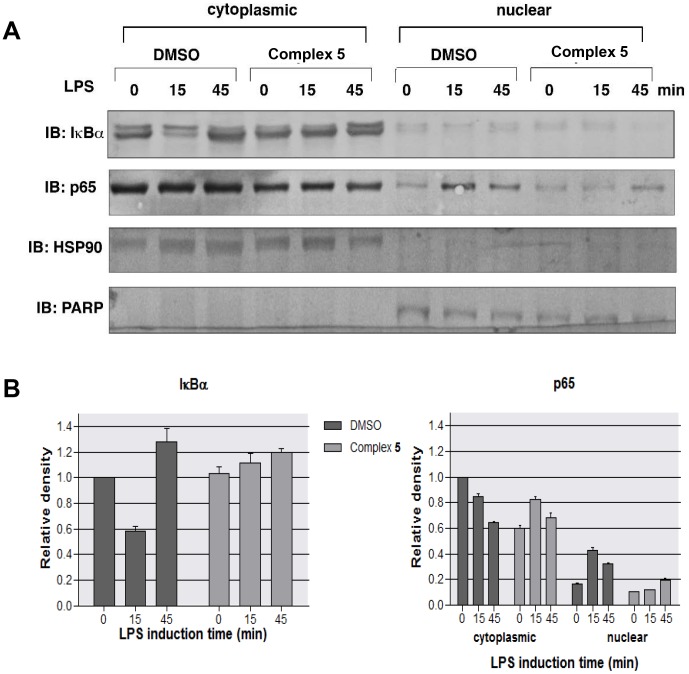
Complex 5 blocks LPS-induced NF-κB nuclear translocation by inhibiting IκBα degradation. (A) RAW264.7 cells pretreated with or without complex **5** for 4 h were treated with LPS for the indicated time points. Cytoplasmic and nuclear extracts were prepared and were subjected to Western blot analysis with IκBα, p65, HSP90 and PARP antibodies. (B) Relative density of IκBα and p65. Western blot signal intensities were quantified using ImageJ software. Densities were normalized to 0 min DMSO.

Next, complex **5** was chosen for subsequent mechanistic study due to its potent inhibitory effect on NF-κB p65 nuclear translocation. In the absence of stimulant, IκBα forms a complex with NF-κB p65 in the cytoplasm. Upon activation by LPS, the degradation of IκBα enables nuclear translocation of p65. To examine the status of IκB in the presence of complex **5**, we pretreated RAW264.7 cells with complex **5** or DMSO for 4 h before incubating with LPS for various time periods. Cytoplasmic and nuclear extracts were separated and analyzed with immunoblotting using anti-IκBα and anti-p65 antibodies (Figure 4A and 4B). After 15 min of LPS stimulation, we observed degradation of IκBα in the cytoplasm. This occurred in conjunction with the translocation of p65 into the nucleus in control cells. Pre-treating the cells with complex **5** inhibited both IκBα degradation and p65 nuclear translocation in response to LPS stimulation. The separation of cytoplasmic and nuclear extracts was verified with immunoblotting using antibodies against PARP (a nuclear protein) and HSP90 (a cytoplasmic protein). Taken together, these results suggest that complex **5** attenuates LPS-induced activation of NF-κB by inhibiting IκBα degradation.

### Complex 5 inhibits TNFα-induced NF-κB translocation in HeLa S3 cells

**Figure 5:**
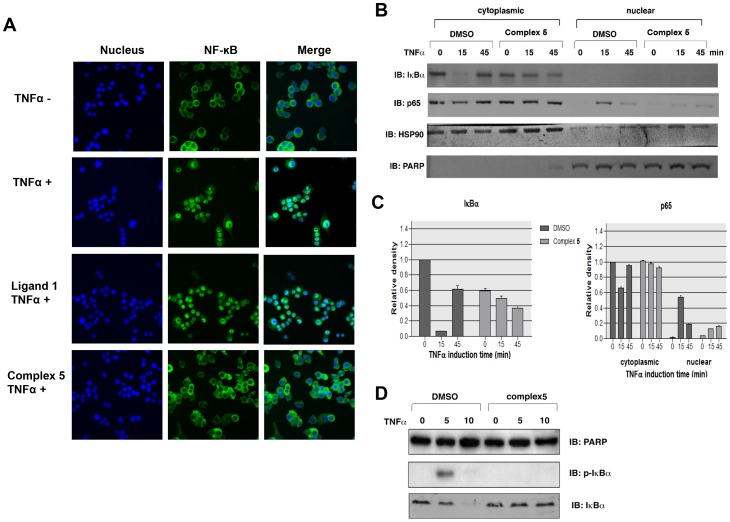
Complex 5 blocks TNFα-induced NF-κB nuclear translocation. Complex **5** inhibits TNFα -induced NF-B nuclear translocation. (A) NF-κB activation assay on TNFα treated HeLa S3 cells. Staining of Hoechst (nucleus) and NF-κB in HeLa S3 cells. Cells were pre-treated with 25 µg/ml of Ligand **1** and Complex **5** for 4 h, followed by TNFα stimulation for 1 h. (B) HeLa S3 cells pretreated with or without complex **5** for 4 h were incubated with TNFα for the indicated time points. Cytoplasmic and nuclear extracts were prepared and were subjected to western blot analysis with IκBα, p65, HSP90 and PARP antibodies. (C) Relative density of IκBα and p65. Western blot signal intensities were quantified using ImageJ software. Densities were normalized to 0 min DMSO. (D) HeLa S3 cells pretreated with or without complex **5** for 4 h were incubated with TNFα for the indicated time points. Whole cell extracts were prepared and were subjected to western blot analysis with IκBα, p-IκBα and PARP antibodies.

To examine whether complex **5** blocks the activation of NF-κB by another agonist, similar experiments were performed on HeLa S3 cells induced with TNFα. Similar to LPS-treated RAW264.7 cells, TNFα-induced translocation of NF-κB p65 in HeLa S3 cells (Figure 5A). Pretreatment of HeLa S3 cells with a very low concentration of complex **5** (6.25 µg/ml) significantly blocked TNF-induced translocation of NF-κB. Moreover, biochemical fractionation showed that pre-treating HeLa S3 cells with complex **5** dramatically reduced IκBα degradation and p65 translocation in response to TNFα stimulation (Figure 5B and 5C). In addition, we found that TNF-induced phosphorylation of IκBα was inhibited by complex **5** (Figure 5D). TNF stimulation induced a rapid phosphorylation of IκBα in DMSO treated cells (within 5 minutes). Pretreating cells with complex **5** completely abolished TNF-mediated phosphorylation of IκBα, thus protecting IκBα from TNF-induced degradation.

### Complex 5 blocks NF-κB transactivation activity

**Figure 6:**
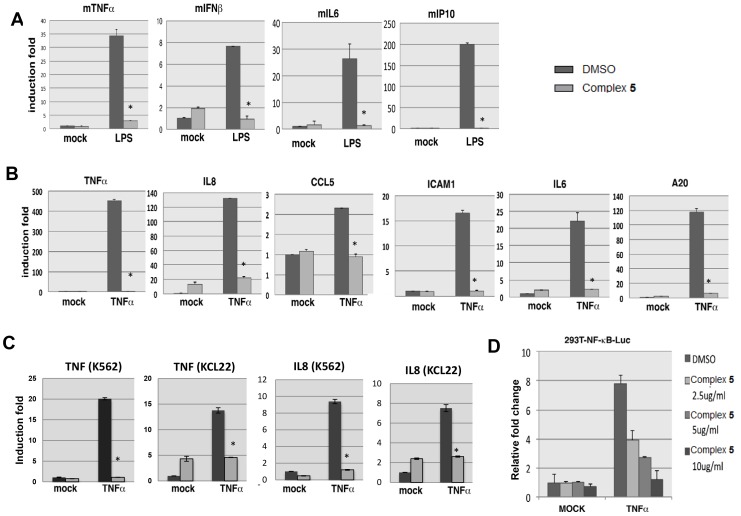
Complex 5 inhibits NF-κB transactivation activity. (A) RAW264.7 cells pretreated with or without complex **5** for 4 h were treated with LPS for 4 h. The expression of TNFα, IFNβ, IL6 and IP10 were measured by QPCR. (B) HeLa S3 cells pretreated with or without complex **5** for 4 h were stimulated with TNFα for 4 h. The expression of TNFα, IL8, CCL5, ICAM1, IL6 and A20 were measured by QPCR. (C) K562 or KCL22 cells pretreated with or without 50 µg/ml of complex **5** for 4 h were stimulated with 100 ng/ml TNFα for 2 h. The expression of IL8 and TNF were measured by QPCR. (D) 293T-luc reporter cells pretreated with or without various concentrations of complex **5** were stimulated with TNFα for 12 h. Cell lysates were prepared and the luciferase activities were measured. Error bars represent the variation range of duplicate experiments. Asterisks represents statistical significance by Student's *t*-test (*P<0.05).

To test if complex **5** inhibits NF-κB-mediated gene expression, we cultured RAW264.7 cells with or without complex **5** for 4 h before treating the cells with LPS for 4 h. Cells were collected for RNA isolation and real time PCR analysis (Figure 6A). Our results showed that LPS-induced expression of NF-κB target genes, TNFα, IFNβ, IL6 and IP10 was abolished following pretreatment of the cells with complex **5**.

In addition, we investigated the effect of complex **5** on NF-κB-mediated gene expression in response to TNFα stimulation in human HeLa S3 cells. We treated HeLa S3 cells with complex **5** for 4 h before TNFα treatment for 4 h. A marked decrease in the expression of NF-κB target genes, including IL8, TNFα, IL6, ICAM1, CCL5 and A20, was observed in complex **5**-treated cells compared to control (Figure 6B). Similar results were obtained in two CML cell lines, K562 and KCL22 (Figure 6C). Furthermore, we showed that complex **5** inhibited TNF-induced expression of COX-2 in a dose dependent manner (Supporting Information Figure S1). Taken together, these results suggest that complex **5** attenuates both LPS- and TNFα-induced transcription of NF-κB regulated genes associated with inflammatory response.

To examine whether complex **5** could inhibit NF-κB transactivation activity, we performed luciferase reporter assay using 293T-luc cell line stably transduced with a promoter containing NF-κB elements-driven luciferase reporter gene (Figure 6D). The cells were pretreated with complex **5** for 4 h, followed by TNFα for 12 h before measuring the luciferase activities. Our data showed that the luciferase activity was induced 8-fold by TNFα stimulation in control cells. Whereas treating the cells with complex **5** significantly reduced the luciferase activity in a dose-dependent manner (Figure 6D), indicating that complex **5** efficiently inhibits NF-κB transactivation activity.

### Complex 5 suppresses acute inflammation *in vivo*

Next, we examined the anti-inflammatory effects of complex **5** in a carrageenan-induced paw edema mouse model. We first tested the acute toxicity effect of complex **5** in C57BL/6 mice. All tested mice displayed no overt abnormalities during a 14 day observation period. Renal and liver functional tests suggested that a low amount of complex **5** up to 125 mg/kg or 250 mg/kg was safe for consumption, but a higher concentration at 500 mg/kg may be cytotoxic to the host (Supporting Information Table S2 and S3).

**Figure 7:**
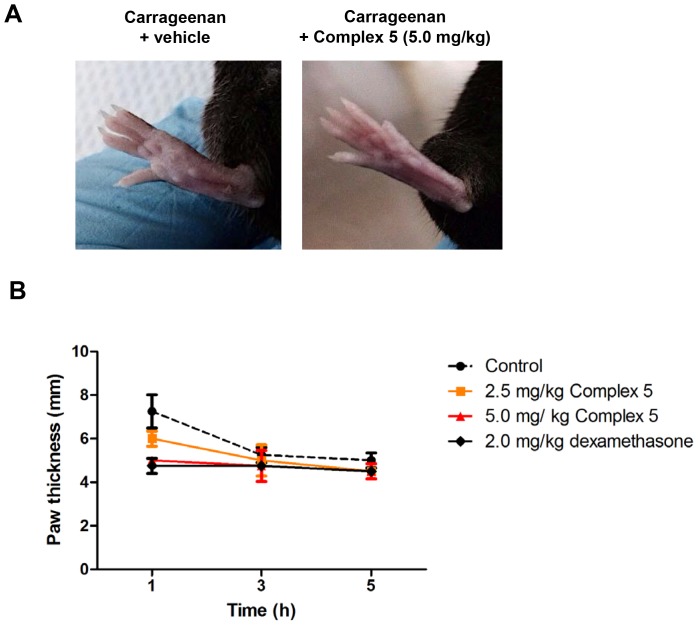
Complex 5 suppresses acute inflammatory responses *in vivo*. Male C57BL/6 mice at age of 8-12 weeks old were non-injected or injected i.p. with 2.5 mg/kg or 5.0 mg/kg complex 5, whereas for positive controls, mice were injected i.p. with 2.0 mg/kg dexamethasone. After 1 h, acute inflammation was induced in the right hind paw of each mice by injecting 50 µl of carrageenan. The size of the paw were measured after (A) 1 h or, (B) 1, 3 and 5 h post carrageenan injection. Shown were mean ±SD (n = 4). Statistical analysis was performed with Student's *t*-test. At 1 h post-carrageenan induction, control versus 2.5 mg/kg complex **5** (P<0.05), control versus 5.0 mg/kg complex **5** (P<0.01), and control versus 2.0 mg/kg dexamethasone (P<0.01).

To test the *in vivo* inhibitory effect of complex **5** on inflammation, we administrated two doses of complex **5** (2.5 mg/kg and 5.0 mg/kg) into C57BL/6 wild type mice through i.p. injection. For a positive control, we performed i.p. injection of 2.0 mg/kg dexamethasone into the mice. After 1 h, paw edema in the mice was induced by injecting 1% carrageenan to the right hind paw. The paw sizes were measured at 1, 3 and 5 h after edema induction. The edema formation at the mice paw was due to inflammatory response causing the accumulation of histamine, serotonin, prostaglandin and other secreted inflammatory mediators. From visual observation, the group of mice administrated with 5.0 mg/kg complex **5** demonstrated obvious reduction of paw edema formation (Figure 7A). One hour after induction of inflammation, paw size in control mice increased to 7.3±0.8 mm. In mice preinjected with 2.5 mg/kg or 5.0 mg/kg of complex **5**, we observed a reduction of paw size to 5.8±0.4 mm and 5.0±0.2 mm, respectively. This was comparable to the paw size (4.6±0.4 mm) in mice pretreated with standard drug, dexamethasone (Figure 7B). At 3 and 5 h post carrageenan administration, paw edema was further reduced in complex **5**-treated mice compared to untreated mice, supporting the ability of complex **5** to suppress acute inflammation *in vivo*.

### Complex 5 does not function as an anti-oxidant

The association between inflammation and reactive oxygen species (ROS) has been demonstrated by a previous study [34]. ROS may amplify inflammatory responses via up-regulation or activation of certain genes/transcription factors, such as NF-κB. Several reports have suggested the possible antioxidant capacity of some transition metals such as copper and iron [35], [36]. Therefore, we conducted ORAC assays to investigate the antioxidant capacity of complex **5**. The ORAC assay uses peroxyl radical as pro-oxidant and the antioxidant activity is quantified via area under curve (AUC) [37]. We included quercetin, a known antioxidant, as a positive control. We found that complex **5**, as well as the other nickel-containing complexes synthesized exhibited low antioxidant activity compared to quercetin (Table 4), indicating that these compounds have little or no antioxidant activity. Therefore, we suggest that complex **5** does not function as an antioxidant as it is not a potent ROS scavenger.

**Table 4:** ORAC assay.

Complex	µM TE per 100 µM
H_3_L^1^ (**1**)	5.18±0.05
H_3_L^2^ (**2**)	8.23±0.43
H_3_L^3^ (**3**)	9.71±0.77
H_3_L^4^ (**4**)	7.54±1.61
[Ni(H_2_L^1^)(PPh_3_)]Cl (**5**)	10.56±0.17
[Ni(H_2_L^2^)(PPh_3_)]Cl (**6**)	15.65±0.89
[Ni(HL^3^)(PPh_3_)] (**7**)	9.37±1.14
[Ni(HL^4^)(PPh_3_)] (**8**)	13.34±1.49
Quercetin	24.41±0.35

Antioxidant capacity of Ni(II) thiosemicarbazone ligands and their Ni(II)-phosphine complexes by ORAC assay.

### Complex 5 docks to active site of IKKβ

**Figure 8:**
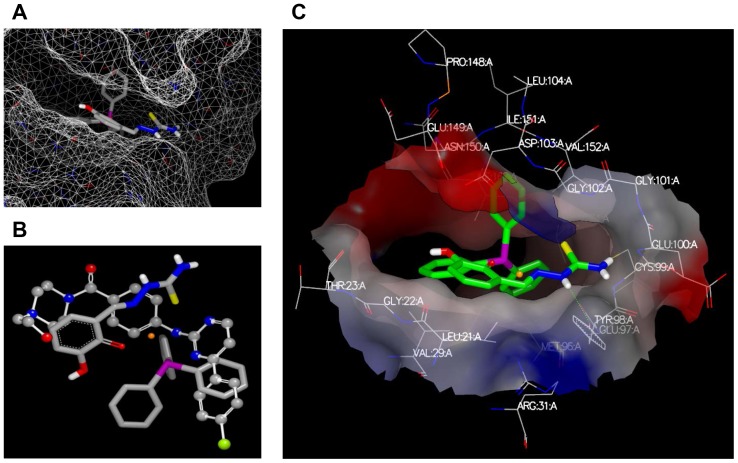
Molecular docking of complex 5 to IKK complex. (A) The binding of complex **5** in the kinase domain of IKKβ. The protein is represented by the protein surface and complex **5** in a stick model. (B) The overlay of complex **5** and inhibitor (compound 1 [30]). The inhibitor is represented by a balls and sticks model and complex **5** by a sticks model, in which the Nickel is colored in orange, nitrogen is colored in blue, oxygen is in red, carbon is in grey, sulphur is in green, hydrogen in white and phosphate is in purple. (C) The interaction between complex **5** and the binding site. One hydrogen bond is formed between complex **5** and TYR98, which is indicated by a green dotted line.

To further understand the mechanism of complex **5** mediated inhibition of NF-κB activation, we reasoned that degradation of IκB to activate NF-κB can be achieved by the upstream activation of IKK kinases [14]. To give some further clues as to whether complex **5** might inhibit IKK kinases, complex **5** was docked to IKKβ, for which the crystal structure is available [31]. Complex **5** was successfully docked into the kinase domain of IKKβ and the triphenylphosphine docked deep into the active site (Figure 8A). The alignment of complex **5** and a previously known inhibitor [31], showed a good overlay and potentially mimics the molecular interactions of the inhibitor (Figure 8B). Based on this docking pose (Figure 8C), we can speculate that the triphenylphosphine is “locked” deeply into the binding site and have hydrophobic interactions with surrounding residues. The hydrophilic side chain of complex **5** points outside the binding site and forms one hydrogen bond with TYR98. This docking pose gives a possible explanation as to why those compounds without triphenylphosphine have poor biological activities. Further, complexes **6** to **8** contain larger hydrophobic side chains that would point towards the solvent accessible area, which may preclude binding in a similar pose.

## Discussion

In the present study, we synthesized four thiosemicarbazones derivatives of 2,3-dihydroxybenzaldehyde, differing in the substituent on N4 of the thiosemicarbazone (H; H_3_L^1^, CH_3_; H_3_L^2^, C_6_H_5_; H_3_L^3^ and C_2_H_5_; H_3_L^4^). We have described the synthesis, characterization and anti-inflammatory activities of Ni(II) thiosemicarbazone complexes with incorporated triphenylphosphine as an auxiliary ligand. Results from spectroscopic data and crystal structure analysis showed that these four complexes are coordinated with the ONS tridentate thiosemicarbazone ligand bounded to Ni(II) through phenolic oxygen, azomethine nitrogen and thiolate/thione sulfur. Of note, we found that the N4-substituted groups on the thiosemicarbazone moiety may influence both the coordination mode of thiosemicarbazone to Ni(II) and their inhibitory effect on NF-κB translocation.

The biological activities of the Ni(II) complexes are possibly influenced by the type of N4 substituents as well as the presence of co-ligands. Our findings suggested that ligands **1** to **4** demonstrated very little effect on the blockage of LPS-mediated nuclear translocation of NF-κB in RAW264.7 cells compared to complexes **5** to **8**, indicating the importance of co-ligands in mounting the inhibitory activity. Among the synthesized complexes **5**, **6**, **7** and **8**, interestingly, only complex **5** showed a marked inhibition of NF-κB translocation. The inhibitory activity of complex **5** could be related to the square planar geometry, which is coordinately unsaturated. Further details on the compounds were as provided (Supporting Information Discussion S1). Based on the findings, we observed that the inhibition efficiency of Ni(II) complexes with 2,3-dihydroxybenzaldehyde N4- thiosemicarbazone in the presence of auxiliary ligand can be arranged according to the substituents on N4 of the thiosemicarbazone moiety as phenyl < C_2_H_5_ < CH_3_ <H. The higher inhibition efficiency by complex **5** may be attributed to the less steric hindrance between the unsubstituted N4- thiosemicarbazone with its potential interactive target such as IKKβ as demonstrated in this study.

MTT cell viability assays showed that compounds **1–8** were relatively less cytotoxic to RAW264.7 and HeLa S3 cells (IC_50_>15 µg/ml), as compared to the US National Cancer Institute recommended guideline for pure compound (IC_50_<4 µg/ml) [38]. In fact, as low as 6.25 µg/ml of complex **5** is sufficient to prevent TNFα-induced NF-κB nuclear translocation after 4 h treatment in HeLa S3 (cell viability still remained >90%), indicating that the inhibition effect was not due to cell death or apoptosis. Importantly, complex **5** did not showed significant cell growth inhibition on primary Human Umbilical Vein Endothelial Cells (HUVEC), with IC_50_>80 µg/ml, indicating less cytotoxicity towards normal cells. Further acute toxicity test in C57BL/6 mice showed that complex **5** could be well tolerated up to 250 mg/kg. Of note, we observed that 5 mg/kg of complex **5** effectively reduced carrageenan-induced paw edema formation, supporting the notion that complex **5** is a potential anti-inflammatory agent, consistent with the *in vitro* experiments using cell lines.

In response to inflammatory stimulants, such as LPS and TNFα, cells could mount immune responses through the activation of NF-κB, a key transcription factor that regulates innate and adaptive immune responses. NF-κB is a transcription factor whose activity is regulated mainly by its nuclear translocation. In resting cells, NF-κB is sequestered in the cytoplasm through its association with IκBα. In response to stimuli, such as proinflammatory cytokines and pathogen-associated molecules, IκBα is phosphorylated by IKKβ and degraded by ubiquitin-proteasome. Degradation of IκBα released NF-κB and enabled its translocation into the nucleus to activate multiple inflammatory-associated genes [14]. Among the NF-κB target genes examined, TNFα, IFNβ, IL6 and IL1β are pro-inflammatory cytokines that exacerbates inflammatory responses. CCL5 (Rantes), IL8 (CXCL8), and IP10 (CXCL10) functions as chemoattractant for other immune cells while ICAM is ligand for LFA-1 integrin that promotes leukocyte adhesion to endothelial cells and transmigration into tissue. A20 (TNFAIP3) is a ubiquitination-associated protein which plays an important role in the NF-κB pathway activation. Our study showed that complex **5** treatment could block NF-κB nuclear translocation and attenuate the expression of these proinflammatory genes after TNFα or LPS stimulation, further supporting its potential role as an anti-inflammatory agent.

To understand the underlying inhibitory mechanism, we performed cytoplasmic/nuclear fractionation and Western blotting. Results showed that both TNFα- and LPS-induced IκBα protein degradation was blocked in the presence of complex **5**, suggesting that complex **5** impaired the upstream signaling pathway that involves the IKK kinase complex. Activated IKK complex phosphorylates and promotes the degradation of IκB, thus enables nuclear translocation of NF-κB. Using molecular docking, we demonstrated potential interactions between complex **5** and the active site of IKKβ in a manner that mimics the interactions with a known inhibitor [31]. In this position, the triphenylphosphine appears to bind deep with the hydrophilic side chain of complex **5** pointing outside the binding site, forming one hydrogen bond with TYR98, which gives a possible explanation as to why those compounds without triphenylphosphine or that contain larger hydrophobic side chains have poor biological activities. Thus, we propose that interaction of IKKβ with complex **5** could reduce LPS- or TNFα-mediated IκBα degradation, which subsequently blocked NF-κB nuclear translocation.

In conclusion, we found that complex **5** efficiently blocked TNF- or LPS-induced NF-κB nuclear translocation compared to other tested compounds. This resulted in lower transcriptional activity, as reflected by the downregulation of pro-inflammatory cytokines mRNA levels. Further *in vivo* studies showed that complex **5** could suppress acute inflammation in mice. As a pilot study, molecular docking was used to predict complex **5** binding at the active site of IKKβ, which could explain how complex **5** might interrupt the LPS or TNFα-induced IKK/IκBα/NF-κB activation pathway.

## Supporting Information

Figure S1 **Complex 5 inhibits TNF-induced COX2 expression.**
(PDF)

Table S1 **Sequences of the primers used in quantitative PCR analysis.**
(PDF)

Table S2 **Renal functional test.**
(PDF)

Table S3 **Liver functional test.**
(PDF)

Discussion S1 (PDF)
