# Supplementary material for: Nickel(II) Complex of Polyhydroxybenzaldehyde N4-Thiosemicarbazone Exhibits Anti-Inflammatory Activity by Inhibiting NF-κB Transactivation
Source: PLoS One. 2014 Jun 30;9(6):e100933. doi: 10.1371/journal.pone.0100933 (PMC4076215; doi:10.1371/journal.pone.0100933)

Supporting Information

Figure S1. Complex **5** inhibits TNF-induced COX2 expression. HeLa S3 cells pretreated with or without various concentrations of complex **5** as indicated for 4 h were stimulated with TNFα for 2 h. The expression of COX2 and TNF were measured by QPCR.

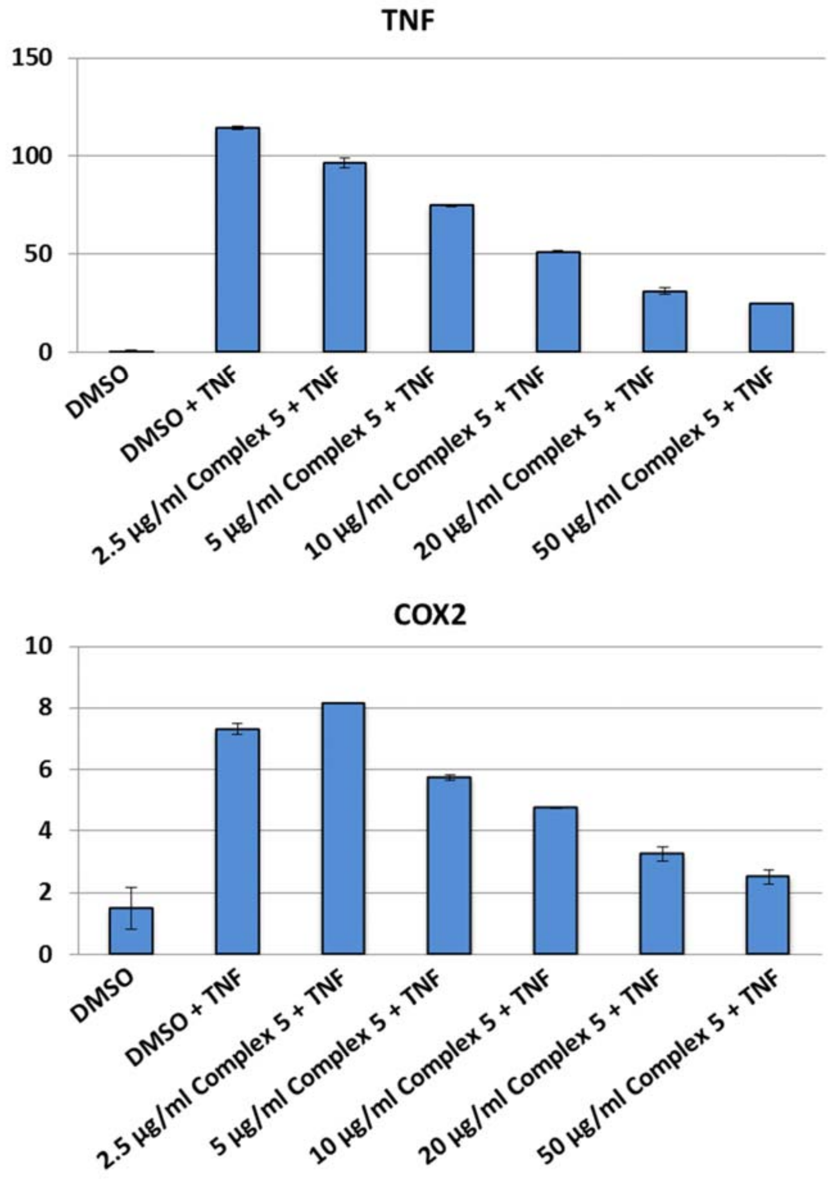

Supplement: Figure S1 — Complex 5 inhibits TNF-induced COX2 expression. (PDF) [file pone.0100933.s001.pdf]
