# Supplementary material for: Nickel(II) Complex of Polyhydroxybenzaldehyde N4-Thiosemicarbazone Exhibits Anti-Inflammatory Activity by Inhibiting NF-κB Transactivation
Source: PLoS One. 2014 Jun 30;9(6):e100933. doi: 10.1371/journal.pone.0100933 (PMC4076215; doi:10.1371/journal.pone.0100933)
Supplement: Table S1 — Sequences of the primers used in quantitative PCR analysis. (PDF) [file pone.0100933.s002.pdf]

Table S1

Sequences of the primers used in quantitative PCR analysis.

Primers	Sequences
hL32 5’	AGCTCCCAAAAATAGACGCAC
hL32 3’	TTCATAGCAGTAGGCACAAAGG
hTNFα 5’	CTATCTGGGAGGGGTCTTCC
hTNFα 3’	ATGTTCGTCCTCCTCACAGG
hIL-8 5’	AGCTCTGTCTGGACCCCAAG
hIL-8 3’	GAATTCTCAGCCCTCTTCAAAAAC
hCCL5 5'	CCCAGCAGTCGTCTTTGTCA
hCCL5 3'	TCCCGAACCCATTTCTTCTCT
hICAM-1 5'	TCTGTGTCCCCCTCAAAAGTC
hICAM-1 3'	GGGGTCTCTATGCCCAACAA
hA20 5'	AAGCTGTGAAGATACGGGAGA
hA20 3'	CGATGAGGGCTTTGTGGATGAT
hIL-6 5'	AAATTCGGTACATCCTCGACGG
hIL-6 3'	GGAAGGTTCAGGTTGTTTTCTGC
hCOX2 5'	TGAGCATCTACGGTTTGCTG
hCOX2 3'	TGCTTGTCTGGAACAACTGC
mL32 5’	AACCCAGAGGCATTGACAAC
mL32 3’	ATTGTGGACCAGGAACTTGC
mTNFα 5’	CTACTCCCAGGTTCTCTTCAA
mTNFα 3’	GCAGAGAGGAGGTTGACTTTC
mIFNβ 5’	CCCTATGGAGATGACGGAGA
mIFNβ 3’	CTGTCTGCTGGTGGAGTTCA
mIL-6 5'	CCGGAGAGGAGACTTCACAG
mIL-6 3'	TCCACGATTTCCCAGAGAAC
mIP-10 5'	AGGACGGTCCGCTGCAA
mIP-10 3'	CATTCTCACTGGCCCGTCAT
