# Supplementary material for: Nickel(II) Complex of Polyhydroxybenzaldehyde N4-Thiosemicarbazone Exhibits Anti-Inflammatory Activity by Inhibiting NF-κB Transactivation
Source: PLoS One. 2014 Jun 30;9(6):e100933. doi: 10.1371/journal.pone.0100933 (PMC4076215; doi:10.1371/journal.pone.0100933)
Supplement: Table S2 — Renal functional test. (PDF) [file pone.0100933.s003.pdf]

Table S2

Renal functional test. C57BL/6 mice at age 8-12 weeks old were administrated orally with control PBS or with various concentrations (125 mg/kg, 250 mg/kg and 500 mg/kg) of complex **5**. n= 6 (3 male and 3 female mice)/group. After two weeks, sera were collected from the mice for renal functional test. Note that there were no significant changes observed for sodium, potassium, chlorine, urea and creatinine levels in mice sera except a slight changes in the CO_2 and anion gap levels. Shown were mean ± SD.

	**Sodium (mmol/L)**	**Potassium (mmol/L)**	**Chlorine (mmol/L)**	**CO_2 (mmol/L)**	**Anion Gap (mmol/L)**	**Urea (mmol/L)**	**Creatinine (umol/L)**
Control	153.25 ± 0.95	5.75 ± 0.55	114.75 ± 1.50	10.85 ± 2.78	33.75 ± 1.89	9.95 ± 1.03	<9
125 mg/kg	147.00 ± 1.22	6.68 ± 1.12	110.80 ± 1.30	18.18 ± 1.37	24.80 ± 0.83	10.72 ± 1.14	<9
250 mg/kg	147.50 ± 1.04	6.50 ±0.52	111.00 ± 1.78	16.36 ± 2.54	26.67 ± 2.25	9.96 ± 1.15	<9
500 mg/kg	148.33 ± 1.86	5.83 ± 0.74	110.83 ± 1.60	16.14 ± 2.08	27.00 ± 1.41	9.56 ± 1.44	<9
