# Supplementary material for: Nickel(II) Complex of Polyhydroxybenzaldehyde N4-Thiosemicarbazone Exhibits Anti-Inflammatory Activity by Inhibiting NF-κB Transactivation
Source: PLoS One. 2014 Jun 30;9(6):e100933. doi: 10.1371/journal.pone.0100933 (PMC4076215; doi:10.1371/journal.pone.0100933)
Supplement: Table S3 — Liver functional test. (PDF) [file pone.0100933.s004.pdf]

Table S3

Liver functional test. C57BL/6 mice at age 8-12 weeks old were administrated orally with control PBS or with various concentrations (125 mg/kg, 250 mg/kg and 500 mg/kg) of complex **5**. n= 6 (3 male and 3 female mice)/group. After two weeks, sera were collected from the mice for liver functional test. Note that for most of tests performed (total protein, albumin, total and conjugated bilirulin, and G-glutamyl transferase) showed no marked changes between control mice and of complex **5**-treated mice. However, some parameters, such as ALP level were elevated from 163.00 ± 7.18 IU/L in the control mice to 219.50 ± 4.95 IU/L in mice treated with a high dose of complex 5 at 500 mg/kg. On the contrary, a marked reduction of AST level from control 453.00 ± 76.29 to 316.50 ± 78.49 were observed in mice treated with high dose of Complex 5 at 500 mg/kg. Shown were mean ± SD. TBIL: Total Bilirubin, BC: Conjugated Bilirubin, ALP: Alkaline Phophatase, ALT: Alanine Aminotransferase, AST: Aspartate Transaminase, GGT: G-Glutamyl Transferase.

	**Total Protein (g/L)**	**Albumin (g/L)**	**Globulin (g/L)**	**TBIL (umol/L)**	**BC (umol/L)**	**ALP (IU/L)**	**ALT (IU/L)**	**AST (IU/L)**	**GGT (IU/L)**
Control	59.00 ± 0.97	17.00 ± .070	42.00 ± 0.37	3.00 ± 0.00	<1	163.00 ± 7.18	91.00 ± 3.06	453.00 ± 76.29	<3
125 mg/kg	60.00 ± 2.12	18.00 ± 0.57	42.00 ± 1.06	3.00 ± 0.45	<1	192.00 ± 3.52	69.00 ± 7.11	421.00 ± 40.81	<3
250 mg/kg	59.00 ± 1.08	17.00 ± 1.41	42.00 ± 0.00	2.50 ± 0.70	<1	195.00 ± 5.66	70.00 ± 4.10	414.00 ± 79.20	<3
500 mg/kg	53.50 ± 0.70	16.00 ± 1.41	37.50 ± 2.12	3.00 ± 0.00	<1	219.50 ± 4.95	70.00 ± 8.49	316.50 ± 78.49	<3
