# Supplementary material for: Nickel(II) Complex of Polyhydroxybenzaldehyde N4-Thiosemicarbazone Exhibits Anti-Inflammatory Activity by Inhibiting NF-κB Transactivation
Source: PLoS One. 2014 Jun 30;9(6):e100933. doi: 10.1371/journal.pone.0100933 (PMC4076215; doi:10.1371/journal.pone.0100933)
Supplement: Discussion S1 — (PDF) [file pone.0100933.s005.pdf]

Supporting Information Discussion

The coordination of thiosemicarbazone ligands with Ni(II) was confirmed by comparing IR spectra of the free ligands with those of the complexes. The chelation of the thiosemicarbazone ligands can be easily detected by monitoring the position of the ligand bands which have shifted to lower or high energy upon complexation. Absorption due to phenolic O-H still can be observed in the IR spectra of all the complexes at around 3450 - 3400 cm^{-1} due to polyhydroxy groups in the thiosemicarbazone ligands. This indicates that only one of the phenolic oxygen atoms from each thiosemicarbazone ligand is deprotonated and involved in coordination with Ni(II) [1,2]. In addition, the band appeared in the region 1307–1270 cm^{-1} due to phenolic C–O stretching in the free thiosemicarbazone ligands has been downward shifted to 1268-1230 cm^{-1} in the IR spectra of the complexes indicating the coordination through phenolic oxygen atom [3]. The involvement of phenolic oxygen in bonding is supported by the appearance of a new band for ν (Ni–O) at 520-510 cm^{-1} region [4]. In all complexes, the ν(C=N) underwent a change in frequency and intensity, caused by complexation and upshifted by 20-50 cm^{-1} to higher frequency. A number of thiosemicarbazones which undergo a similar shift of the ν(C=N) bands to higher wave numbers upon coordination has also been reported [5]. The coordination of azomethine nitrogen to Ni(II) is further corroborated with the appearance of a new band at around 565 - 532 cm^{-1} region due to a ν(Ni—N) stretch in the spectra of all complexes. However, the coordination of thiosemicarbazone sulfur to Ni(II) either in its thione or thiol form was confirmed by the downward shift of 10-80 cm^{-1} for υ(C=S) in the spectra of all complexes. Additionally, the IR spectra of all complexes show bands characteristic of coordinated triphenylphosphine in the expected region [6-8].

Proton magnetic resonance spectroscopy is a helpful tool for the identification of the coordination sites in diamagnetic Ni(II) complexes in conjunction with other spectrometric information. The singlet appeared in the region 11.35 – 11.77 ppm in the 1HNMR spectra free ligands, which assigned to the N(2)HCS group, is absent in the spectrum of complexes 7 and 8 indicating the coordination of the ligand under the anionic thiolate form upon deprotonation at N(2). In the spectra of complex **5** and

complex **6**, the hydrazinic proton N(2)H signal appeared at 11.45 ppm and 11.43 ppm, respectively. The slightly downfield shift of this signal suggested the coordination of the ligand to the metal center in the neutral thione form [9]. The azomethine proton –CH=N does not show a regular trend in the complexes. The azomethine –CH=N signals which observed at 8.33–8.52 ppm in the free thiosemicarbazone undergoes shift to the high field, thus, indicating coordination of the thiosemicarbazone through the imine nitrogen chelating system. In contrast, the azomethine proton in the complexes with N(4)–phenyl substitution (i.e. complex **7**), is shifted downfield due to the presence of an electron withdrawing phenyl group [10,11]. The dihydroxybenzaldehyde aromatic protons are shifted to the high field environment due to the decrease in the electron density around the thiosemicarbazone moiety as a result of coordination to Ni(II) through sulfur, azomethine nitrogen and phenolic oxygen. It has been illustrated that the decrease in electron density cause an upfield shift of the aromatic protons, different from aliphatic protons, due to electron withdrawing ability of the metal ion, which in turn decreases the ring current and deshielding of the aromatic protons. For this reason a stronger field is required to take the aromatic protons to resonance [12].

The geometry around the nickel atom in these complexes is a distorted square planar as can be seen from the bond angles. The ligands H_3L^3 and H_3L^4 are coordinated as ONS tridentate binegative anion in their deprotonated form HL^3 and HL^4 as is indicated by the absence of counter ions, through azomethine nitrogen, phenolic oxygen and thiolate sulfur atoms forming stable six and five membered rings. The fourth coordination position is occupied by a P atom from the PPh_3 coligand which is coordinated to Ni *trans* to azomethine nitrogen. In complex **8**, the Ni–N, Ni–P, Ni–S bond lengths of 1.8961(14), 2.2059(5), 2.1488(5), respectively are quite normal as that found in other square planar Ni(II) complexes and Ni–O bond length 1.8510(12) was found to be slightly higher than that of the reported value for other Ni(II) complexes [13,14]. This slight increase in Ni–O bond length may be due to the presence of hydroxyl group. Considering the plane formed by the O1, N1, S1, P1 set of chelating atoms, the nickel atom sits 0.0170 Å away from them. The thiosemicarbazone fragment C7,N1,N2,C8,S1,N3, also is close to planar has a maximum least square plane deviation from the central metal atom (Ni1) by –

0.084 Å and the dihedral angle formed by the thiosemicarbazone moiety with the plane containing coordinating atoms is 6.62(5)º which denotes a coplanarity of the thiosemicarbazone moiety [15]. The angles formed between the nickel ion and the coordination sphere O1, N1, S1, P1 show that the nickel is contained within a slightly distorted square-planar environment with the O(1)– Ni(1)– S(1) of 173.72 (4)º and N(1) –Ni(1)– P(1) of 171.55(4)º deviate from the linearity. The coordination of the ligand to Ni(II) leads to a lengthening of the S1–C8 bond 1.7529(18) Å (increased single-bond character) and a shortening of the neighboring N2–C8 bond 1.299(2) Å (increased double-bond character) and in accordance to coordination of thiosemicarbazone in the thiolate form [16,17]. Higher distortion from ideal square planar geometry is observed for complex **8** in contrast to complex **5** [18], as reflected in the bond parameters around the metal center in both complexes. The high deviation of complex **8** from regular square planar arrangement may be due to the steric hindrance between the bulky triphenylphosphine group and the ethyl group at N(4) position.

References

1. Kobakhidze N, Farfán N, Romero M, Méndez-Stivalet JM, Gabriela Ballinas-López M, et al. (2010) New pentacoordinated Schiff-base diorganotin(IV) complexes derived from nonpolar side chain α-amino acids. Journal of Organometallic Chemistry 695: 1189-1199.

2. Tan KW, Seng HL, Lim FS, Cheah S-C, Ng CH, et al. (2012) Towards a selective cytotoxic agent for prostate cancer: Interaction of zinc complexes of polyhydroxybenzaldehyde thiosemicarbazones with topoisomerase I. Polyhedron 38: 275-284.

3. Muthu Tamizh M, Varghese B, Endo A, Karvembu R (2010) NMR (1D and 2D) and X-ray crystallographic studies of Ni(II) complex with N-(2-mercaptophenyl)-4-methoxysalicylideneimine and triphenylphosphine. Spectrochimica Acta Part A: Molecular and Biomolecular Spectroscopy 77: 411-418.

4. Liu JN, Wu BW, Zhang B, Liu YC (2006) Synthesis and characterization of metal complexes of Cu(II),

Ni(II), Zn(II), Co(II), Mn(II) and Cd(II) with tetradentate Schiff bases. Turkish Journal of Chemistry 30: 41-48.

5. Ali MA, Mirza AH, Chartres JD, Bernhardt PV (2011) Synthesis, characterization and X-ray crystal structures of seven-coordinate pentagonal-bipyramidal zinc(II), cadmium(II) and tin(IV) complexes of a pentadentate N3S2 thiosemicarbazone. Polyhedron 30: 299-306.

6. Carty AJ (1967) Coordination complexes of gallium(III) and indium(III) halides. I. Complexes with triarylphosphines. Canadian Journal of Chemistry 45: 345-351.

7. Karvembu R, Hemalatha S, Prabhakaran R, Natarajan K (2003) Synthesis, characterization and catalytic activities of ruthenium complexes containing triphenylphosphine/triphenylarsine and tetradentate Schiff bases. Inorganic Chemistry Communications 6: 486-490.

8. Lobana TS, Khanna S, Hundal G, Kaur P, Thakur B, et al. (2009) Coinage metal derivatives of salicylaldehyde thiosemicarbazones: Synthesis, structures, bond isomerism and H-bonded networks. Polyhedron 28: 1583-1593.

9. Lobana TS, Bawa G, Castineiras A, Butcher RJ (2007) First example of pyrrole-2-carbaldehyde thiosemicarbazone as tridentate dianion in [Pd(η3-N4,N3,S-ptsc)(PPh3)] complex. Inorganic Chemistry Communications 10: 506-509.

10. Belicchi Ferrari M, Bisceglie F, Pelosi G, Sassi M, Tarasconi P, et al. (2002) Synthesis, characterization and X-ray structures of new antiproliferative and proapoptotic natural aldehyde thiosemicarbazones and their nickel(II) and copper(II) complexes. Journal of Inorganic Biochemistry 90: 113-126.

11. Jayabalakrishnan C, Karvembu R, Natarajan K (2002) Catalytic and antimicrobial activities of new ruthenium(II) unsymmetrical Schiff base complexes. Transition Metal Chemistry 27: 790-794.

12. Stringer T, Chellan P, Therrien B, Shunmoogam-Gounden N, Hendricks DT, et al. (2009) Synthesis and structural characterization of binuclear palladium(II) complexes of salicylaldimine dithiosemicarbazones. Polyhedron 28: 2839-2846.

13. Güveli Ş, Özdemir N, Bal-Demirci T, Ülküseven B, Dinçer M, et al. (2010) Quantum-chemical,

spectroscopic and X-ray diffraction studies on nickel complex of 2-hydroxyacetophenone thiosemicarbazone with triphenylphospine. Polyhedron 29: 2393-2403.

14. Güveli Ş, Ülküseven B (2011) Nickel(II)–triphenylphosphine complexes of ONS and ONN chelating 2-hydroxyacetophenone thiosemicarbazones. Polyhedron 30: 1385-1388.

15. Bon VV, Orysyk SI, Pekhnyo VI, Volkov SV (2010) Square-planar 1:2 Ni(II) and Pd(II) complexes with different coordination mode of salicylaldehyde (4)-phenylthiosemicarbazone: Synthesis, structure and spectral properties. Journal of Molecular Structure 984: 15-22.

16. Ketcham KA, Garcia I, Swearingen JK, El-Sawaf AK, Bermejo E, et al. (2002) Spectral studies and X-ray crystal structures of three nickel(II) complexes of 2-pyridineformamide 3-piperidylthiosemicarbazone. Polyhedron 21: 859-865.

17. Ferrari MB, Capacchi S, Bisceglie F, Pelosi G, Tarasconi P (2001) Synthesis and characterization of square planar nickel(II) complexes with p-fluorobenzaldehyde thiosemicarbazone derivatives. Inorganica Chimica Acta 312: 81-87.

18. Shawish HB, Tan KW, Maah MJ, Ng SW (2010) {2-[(2-Carbamothiolylhydrazin-1-ylidene-[kappa]2N1,S)methyl]-6-hydroxyphenolato-[kappa]O1}(triphenylphosphine-[kappa]P)nickel(II) chloride. Acta Crystallographica Section E 66: m1074.
